# Change in Micronutrient Intake among People with Relapsing-Remitting Multiple Sclerosis Adapting the Swank and Wahls Diets: An Analysis of Weighed Food Records

**DOI:** 10.3390/nu13103507

**Published:** 2021-10-05

**Authors:** Tyler J. Titcomb, Lisa Brooks, Karen L. Smith, Patrick Ten Eyck, Linda M. Rubenstein, Terry L. Wahls, Linda G. Snetselaar

**Affiliations:** 1 Department of Internal Medicine, University of Iowa, Iowa City, IA 52242, USA; Tyler-Titcomb@uiowa.edu (T.J.T.); Lisa-Brooks@uiowa.edu (L.B.); karen-l-smith@uiowa.edu (K.L.S.); 2 Department of Epidemiology, University of Iowa, Iowa City, IA 52242, USA; Linda-Rubenstein@uiowa.edu (L.M.R.); Linda-Snetselaar@uiowa.edu (L.G.S.); 3 Fraternal Order of Eagles Diabetes Research Center, University of Iowa, Iowa City, IA 52242, USA; 4 Institute for Clinical and Translational Science, University of Iowa, Iowa City, IA 52242, USA; Patrick-TenEyck@uiowa.edu

**Keywords:** multiple sclerosis, modified Paleolithic diet, low-saturated fat diet, micronutrients, micronutrient inadequacy

## Abstract

The low-saturated fat (Swank) and modified Paleolithic elimination (Wahls) diets have shown promise for MS symptoms; however, due to their restriction of specific foods, inadequate intake of micronutrients is concerning. Therefore, as part of a randomized trial, weighed food records were collected on three consecutive days and were used to evaluate the intake of micronutrients among people with relapsing remitting MS adapting these diets. After randomization to either the Swank or Wahls diets, diet education and support was provided by registered dietitians at baseline and throughout the first 12 weeks of the intervention. Usual intake of each micronutrient was estimated and then evaluated with the EAR-cut point method. At 12 weeks, the Swank group had significant reductions in the proportion with inadequate intake from food for vitamins C, D, and E, while the Wahls group had significant reductions for magnesium and vitamins A, C, D, and E. However, the proportion with inadequate intake significantly increased for calcium, thiamin, and vitamin B_12_ in the Wahls group and for vitamin A in the Swank group. Inclusion of intake from supplements reduced the proportion with inadequate intake for all micronutrients except calcium among the Wahls group but increased the proportion with excessive intake for vitamin D and niacin among both groups and magnesium among the Swank group. Both diets, especially when including intake from supplements, are associated with reduced inadequate intake compared to the normal diet of people with relapsing remitting MS.

## 1. Introduction

Multiple sclerosis (MS) is a chronic neurodegenerative disease that affects nearly 1 million people in the United States [1]. There is considerable interest among people with MS (pwMS) in alternative therapies such as diet as adjunct therapy to help alleviate symptom burden. Surveys consistently observe that half of pwMS report implementing dietary modifications [2,3] despite receiving little dietary advice at the time of diagnosis [4]. PwMS likely obtain information on diet from internet sources [5], which may be problematic since the current state of evidence does not support recommendations for any specific therapeutic diets [6]. 

Preliminary evidence suggests the low-saturated fat diet developed by Dr. Swank and the modified Paleolithic diet developed by Dr. Wahls are potential adjunct approaches for symptom management [7]. On the basis of an observed association of fat intake, especially from high saturated fat sources, and risk of MS [8], Swank recommended his patients to consume a low saturated fat diet [9], and followed them for up to 50 years [10]. He observed that lower saturated fat intake was associated with fewer exacerbations, increased ability to ambulate, and reduced risk of mortality [11,12]. On the basis of her personal [13] and clinical experience [14], Dr. Wahls recommends a diet based on Paleolithic principals that eliminates specific dietary antigens (gluten, casein, and lectins) and maximizes micronutrient density [15]. Preliminary studies comparing the modified Paleolithic diet to usual diet demonstrated favorable outcomes for fatigue and QoL among individuals with progressive or relapsing-remitting MS (RRMS) [16,17,18]. In a head-to-head comparison, both the low-saturated fat and modified Paleolithic elimination diets were associated with within-group improvements in quality of life and reductions in fatigue, but there were no major differences between the two diet groups [19].

Due to the restriction of specific foods in both the low-saturated fat and the modified Paleolithic diets, risk of micronutrient deficiencies is of concern. Inadequate intake of several micronutrients, such as folate and magnesium, is associated with more severe MS symptoms [20,21]; thus, it is imperative that specialized diets for MS avoid these micronutrient shortfalls. Previously, experimental seven-day menus were created for the low-saturated fat and modified Paleolithic elimination diets. Analysis of these experimental menus suggest that the low-saturated fat diet theoretically met the requirements for all micronutrients except calcium; iron; potassium; and the vitamins D, E, folate, and choline for adult male and female (not pregnant or lactating) life stage groups [22]. Similarly, the modified Paleolithic elimination diet met requirements for all micronutrients except vitamin D, calcium, potassium, and choline for most life stage groups and iron for women of childbearing age [23]. The change from usual diet micronutrient inadequacy among pwMS implementing the low-saturated fat or modified Paleolithic elimination diets has not been reported; thus, the aim of this study was to report micronutrient intakes from food of pwMS consuming these diets.

## 2. Materials and Methods

### 2.1. Participants and Study Design

As part of a 36-week, randomized, parallel-group, single-blinded trial comparing the effect of the low-saturated fat and modified Paleolithic diets on fatigue and quality of life among people with relapsing-remitting MS (RRMS) [19]; 3-day weighed food records and food frequency questionnaires (FFQs) were collected at 12-week intervals. The trial protocol [24] was approved by the University of Iowa Institutional Review Board. Written and informed consent was obtained from all participants, and data safety and management of the trial was monitored by a National Multiple Sclerosis Society (NMSS) data safety monitoring board. This trial is registered at Clinicaltrials.gov identifier: NCT02914964 and followed the Consolidated Standards of Reporting Trials (CONSORT) reporting guidelines [25].

Participants were recruited from within 500 miles of Iowa City, Iowa, and were eligible for enrollment in the study if they (1) had definitive RRMS in accordance with the 2010 McDonald criteria [26], (2) had moderate to severe fatigue (FSS ≥ 4.0), (3) had an ability to walk 25 feet with limited or no support, (4) were not pregnant or planning on becoming pregnant, and (5) were willing to comply with all aspects of the study diets and procedures. Major exclusion criteria included (1) MS-relapse or change in MS medication within the previous 12 weeks prior to the study; (2) body mass index (BMI) < 19 kg/m^2^; (3) severe mental impairment; (4) self-reported adverse reactions to gluten-containing foods; (5) diagnosed conditions including eating disorders, severe psychiatric disorders, celiac disease, kidney stones, heart failure, angina, or liver cirrhosis; and (6) insulin, warfarin, radiation, or chemotherapy use. A complete list of inclusion and exclusion criteria can be found in the published protocol [24].

### 2.2. Study Procedures

After a 12-week observation period to monitor usual diet, participants (*n* = 87) were randomized at baseline to either the modified Paleolithic elimination (Wahls; *n* = 43) or the low-saturated fat (Swank; *n* = 44) diets for 24 weeks. During the first 12 weeks of the intervention, participants received two in-person and five telephone-based nutrition counseling sessions from a registered dietitian (RD). Participants also received personalized emails with feedback on their diet checklists every 4 weeks. After 12 weeks of the intervention period, in-person and telephone counseling sessions were discontinued, but participants were allowed to contact the intervention RD for additional support. In addition, the Wahls group participants were instructed to begin reintroducing nightshade vegetables and spices during weeks 13 through 24. The data presented in this article are from the baseline and first 12-week post-randomization period only to avoid variation in diet caused by the reintroduction of the nightshade vegetables and spices among the Wahls group and to limit bias introduced due to dropouts. Data on participants who completed the first 12 weeks of the intervention (*n* = 39 Wahls and *n* = 38 Swank) was included in the present secondary analysis (Table 1). 

### 2.3. Intervention Diets

The Swank diet restricts saturated fat to ≤ 15 g and provides 20–50 g (4–10 teaspoons) unsaturated fat and four servings each of grains (whole grains preferred) and fruits and vegetables (FV) per day (Table 2). The Wahls diet recommends 6–9 servings of FV and provides 6–12 ounces meat per day according to gender. It excludes all grain, legumes, eggs, and dairy (except for clarified butter or ghee). Nightshade vegetables were also excluded in the Wahls group during the first 12-week period of the intervention and then the study RDs provided guidance to reintroduce nightshades during the second 12-week intervention period. A review of both diets can be found elsewhere [7]. Participants in both groups were instructed to follow their assigned diet ad libitum and were given the following daily supplement regimen: 1 teaspoon cod liver oil, 1000 μg methyl-B_12_, 1000 μg methylfolate, a multivitamin without iron, and 5000 IU vitamin D_3_, the latter of which was adjusted on the basis of serum 25-hydroxyvitamin D levels with a target range of 40 to 80 ng/mL [24]. Adherence to the study diets, defined as being within 20% of recommendations for specific key diet components (≤0.2 grams of gluten or ≤18 grams of saturated fat for the Wahls or Swank diets, respectively), was 80% for the Wahls group and 87% for the Swank group at 12 weeks [19].

### 2.4. Food Records and Analysis

Three weighed food records were collected consecutively on two weekdays and one weekend day at baseline and again at 12 weeks. Food records obtained from included participants were analyzed using Nutrition Data System for Research software (University of Minnesota Nutrition Coordinating Center). To account for the differing bioavailability of folate and folic acid, we used dietary folate equivalents (DFEs) for all analyses. Because tryptophan can partially meet niacin requirements, niacin equivalents (NEs) were calculated as 60 mg tryptophan is equal to 1 mg niacin. Bioconversion of provitamin A carotenoids were accounted for by using retinol activity equivalents (RAEs) for vitamin A, and bioconversion of tocopherols was accounted for by using alpha-tocopherol equivalents for all vitamin E analyses. Mean intake of each micronutrient from food was calculated for each participant and adjusted for age, gender, and corresponding 2007 Harvard Food Frequency Questionnaires (FFQs) values using the National Cancer Institute (NCI) method to estimate usual intake [27]. Usual intake of each micronutrient from food was then compared to the estimated average requirement (EAR) for each life stage group using the EAR-cut point method [28] and combined by weighted means to assess the proportion of each group with inadequate micronutrient intake. Usual intake was also compared to Tolerable Upper Intake Levels (ULs) to determine the proportion with excessive intakes. Because iron requirements are not normally distributed among women of reproductive age, proportion with iron inadequate intake was estimated using a manual probability approach recommended by the National Academy of Medicine (formerly the Institute of Medicine) [29]. Micronutrients without EARs such as choline and potassium were excluded from this analysis.

### 2.5. Statistical Analysis

Fisher’s exact test was used to compare categorical variables between groups at baseline (sex, race, education, MS diagnosis, disease-modifying medications, smoking status, alcohol intake), and generalized linear models were used to compare continuous variables (age, MS duration, BMI) between groups at baseline. The within-group baseline to 12-week changes in inadequate micronutrient intake were compared using one-sample proportion *z*-tests. The 12-week difference between groups in inadequate micronutrient intake was compared with two-sample proportion *z*-tests. All analyses were conducted using two-tailed tests, and significance was concluded when alpha ≤ 0.05.

## 3. Results

### Micronutrient Inadequate Intake

At baseline, the proportion with inadequate intake of micronutrients from food among the Swank group were 96.3% for vitamin D, 50.1% for vitamin E, 47.4% for calcium, 44.6% for vitamin C, 37.7% for magnesium, 23.1% for vitamin B_6_, 21.6% for folate, 20.7% for vitamin A, 17.4% for zinc, 14.0% for vitamin B_12_, 12.6% for iron, 11.5% for thiamin, 8.7% for copper, 5.3% for riboflavin, 3.5% for phosphorus, 2.8% for niacin, and 2.7% for selenium (Table 3). At 12 weeks, the Swank group had significant reductions in the proportion with inadequate intake from food for vitamins C (20.6%; *p* < 0.01), E (32.3%; *p* = 0.03), and D (86.2%; *p* < 0.01). At 24 weeks, the proportion with inadequate intake from food was significantly lower than baseline for vitamins C (24.1%; *p* < 0.01), D (77.6%; *p* < 0.01), and B_6_ (4.8%; *p* < 0.01). The proportion with inadequate intake of vitamin A from food was significantly higher at 24 weeks compared to baseline intake (36.6%; *p* = 0.02). Inclusion of intake from supplements lead to a further reduced proportion with inadequate intake for copper; magnesium; zinc, and the vitamins A, C, D, E, B_6_, and thiamin at 12 and 24 weeks (Table 3), All other micronutrients had non-significant differences at 12 and 24 weeks.

Among the Wahls group, the proportion with inadequate intake of micronutrients from food at baseline were 99.9% for vitamin D, 56.1% for vitamin E, 54.7% for calcium, 50.8% for magnesium, 50.0% for vitamin C, 44.3% for vitamin A, 26.4% for folate, 18.3% for iron, 15.3% for vitamin B_6_, 12.5% for zinc, 6.4% for copper, 6.3% for selenium, 5.7% for thiamin, 5.6% for phosphorous, 5.3% for vitamin B_12_, 5.3% for niacin, and 4.5% for riboflavin (Table 4). At 12 weeks, the Wahls group had significant reductions in the proportion with inadequate intake from food for vitamin A (13.1%; *p* < 0.01), vitamin C (1.5%; *p* < 0.01), vitamin D (86.6%; *p* < 0.01), vitamin E (26.7%; *p* < 0.01), and magnesium (28.5%; *p* < 0.01). The Wahls group also had significant increases in the proportion with inadequate intake from food for calcium (86.5%; *p* < 0.01), thiamin (13.6%; *p* = 0.03), and vitamin B_12_ (17.4%; *p* < 0.01) at 12 weeks. All differences remained significant at 24 weeks compared to baseline; however, the proportion with inadequate intake from food at 24 weeks increased for vitamin C (9.3%; *p* < 0.01) and copper (1.4%; *p* < 0.01) compared to week 12 values. Inclusion of intake from supplements lead to a further reduced proportion with inadequate intake for magnesium; zinc; and the vitamins A, C, D, E, B_6_, riboflavin, thiamin, and folate at 12 and 24 weeks (Table 3). Inadequate intake of calcium remained significantly higher at 12 and 24 weeks compared to baseline among the Wahls group, despite inclusion of intake from supplements. All other micronutrients had non-significant differences at 12 and 24 weeks.

**Figure 1:**
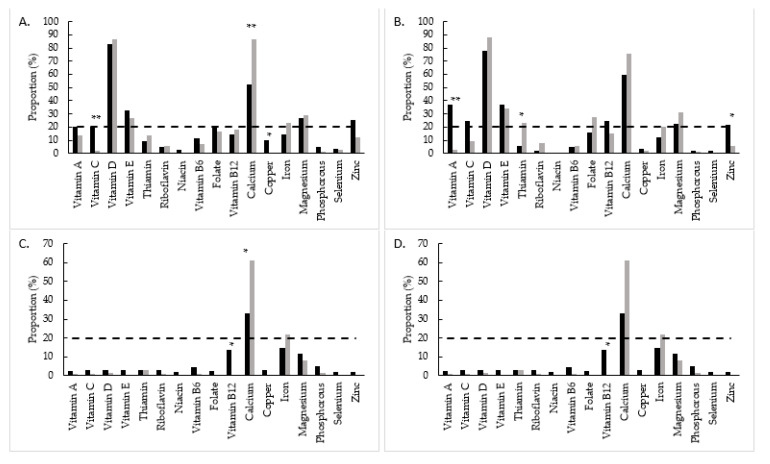
Proportion with inadequate micronutrient intake among the Swank (black bars) and Wahls (grey bars) groups from food at (**A**) 12 weeks and (**B**) 24 weeks and from food and supplements at (**C**) 12 weeks and (**D**) 24 weeks. The dashed line represents severe micronutrient inadequate intake defined as ≥20% of the group. Statistical significance determined by two sample *z*-tests; statistical significance is represented by * = *p* ≤ 0.05 and ** = *p* ≤ 0.01.

Compared to the Swank group, the Wahls group had significantly lower proportion with inadequate intake from food for vitamin C (*p* < 0.01) and copper (*p* = 0.05) at 12 weeks and for vitamin A (*p* < 0.01) and zinc (*p* = 0.04) at 24 weeks (Figure 1A,B). The Swank group had significantly lower proportion with inadequate intake from food for calcium (*p* < 0.01) at 12 weeks and thiamin at 24 weeks compared to the Wahls group. After inclusion of intake from supplements, the Swank group had a higher proportion with inadequate intake of vitamin B_12_ (*p* = 0.02) and lower proportion with inadequate intake of calcium (*p* = 0.02) compared to the Wahls group at both 12 and 24 weeks (Figure 1C,D). There were no other significant differences between groups in the proportion with inadequate intake for other micronutrients at 12 and 24 weeks.

Both groups had a high proportion with excessive intake of vitamin D at baseline (20.8% Swank and 33.9% Wahls), but no other micronutrient was above 10% for either group (Table 5). The proportion of the Swank group with excessive intake increased for vitamin D and niacin at 12 and 24 weeks and for magnesium at 24 weeks, and the proportion with excessive intake of vitamin D increased in the Wahls group at 12 and 24 weeks. All other micronutrients had non-significant differences at 12 and 24 weeks.

## 4. Discussion

The findings from this analysis of 3-day weighed food records show that both the low-saturated fat diet proposed by Swank and the modified Paleolithic elimination diet proposed by Wahls are associated with reduced proportion of or no change in micronutrient inadequate intake compared to the usual diet of people with RRMS. This was with the exceptions of calcium, thiamin, and vitamin B_12_ among the Wahls group, for which the proportion with inadequate intake from food increased significantly; however, only calcium remained increased after the inclusion of supplements.

The usual diet of both groups in this study at baseline were severely inadequate (>20% of the group) in terms of intake of calcium; magnesium; and the vitamins A, C, D, E, and folate. The same micronutrients are also severely inadequately consumed among the general U.S. adult population [30]. Similarly, pwMS have been found to have lower intake of folic acid, magnesium, zinc, and selenium compared to the general Dutch population [31]. These observations are alarming because several studies have found associations between inadequate intake or deficiency of several specific micronutrients and more severe MS symptoms. Cross-sectional studies have shown that diets lowest in folate or magnesium are associated with higher fatigue burden [20] and lower physical ability and quality of life [21]. Furthermore, serum values of vitamins A, D, and E are associated with markers of inflammation [32] and odds of new MRI brain lesions [33,34,35] among pwMS.

In this study, the Swank group had significant reductions in the proportion with inadequate intake from food for vitamins B_6_, C, D, and E compared to their usual diet at baseline. The reduction in inadequate vitamin C intake is likely due to the recommendation for 2+ servings each of fruits and vegetables per day that are rich sources of vitamin C. The reduction in the proportion with inadequate vitamin D intake from food observed among the Swank group is likely due to consumption of vitamin D-fortified foods such as orange juice and non-fat dairy products. However, despite the decreases in the proportion with inadequate intake for vitamins C, D, and E, intake from food for all are still considered severely inadequate at 12 weeks. Similarly, the proportion with inadequate intake from food for folate, calcium, magnesium, and zinc remained above 20% at 12 weeks. However, the proportion with inadequate intake was reduced to below the 20% threshold for all micronutrients except calcium with the inclusion of intake from supplements. These observations are consistent with previous analyses of experimental menus [22] and an online survey of dietary characteristics among pwMS [36]. 

The Wahls group had significant reductions in the proportion with inadequate intake from food for vitamin A, vitamin C, vitamin D, vitamin E, and magnesium at 12 and 24 weeks compared to their usual diet at baseline. The Wahls elimination diet recommends 6–9+ servings per day total fruits and vegetables, 12 ounces per week organ meats, and 4 ounces per day nuts and seeds, which are rich sources of the micronutrients that improved with the diet intervention. However, the Wahls elimination diet recommends strict avoidance of dairy, a rich source of calcium and vitamin B_12_, which likely explains the significant increase in the proportion with inadequate intake for calcium and vitamin B_12_ at 12 weeks. Similarly, the Wahls elimination diet recommends strict avoidance of grains, which are a rich source of thiamin and likely explain the significant increase in the proportion with inadequate intake of thiamin at 12 weeks. The proportion with inadequate intake for calcium, vitamin D, vitamin E, iron, and magnesium all remained above 20% at 12 weeks among the Wahls group, but despite the increased proportion with inadequate intake for thiamin and vitamin B_12_, proportion with inadequate intake from food for these nutrients was above the 20% threshold for severe inadequate intake at 12 weeks, and only calcium and iron were still above 20% after inclusion of intake from supplements. These observations are consistent with analysis of 24-hour dietary recalls from a preliminary trial [37], our previous experimental menu analysis [23], and an online survey of dietary characteristics among pwMS [36]. 

The severe inadequate intake from food of vitamin D, vitamin E, calcium, and magnesium was present among both groups during the intervention, highlighting the importance of supplementation of select micronutrients among people with MS following either the Swank or Wahls diets. Inclusion of intake from supplements reduced the proportion with inadequate intake for most micronutrient shortfalls. In addition to supplementation, pwMS following either diet may benefit from selecting specific micronutrient-dense foods such as sunflower seeds for vitamin E or pumpkin seeds for magnesium. Because both diets restrict or partially restrict dairy, recommendations for the consumption of calcium-fortified foods such as fortified juices or milk alternatives or calcium supplementation may be beneficial. For micronutrients with few rich food sources such as vitamin D, supplementation is recommended for pwMS [38], especially those who are severely disabled, which may prevent adequate time spent outdoors for sun-derived vitamin D [39]. Furthermore, because severe inadequate intake was present at 12 weeks for folate and zinc among the Swank group only, pwMS who follow this dietary strategy may benefit from increased consumption of vegetables and whole grains for folate and low-saturated fat shellfish for zinc. Similarly, pwMS who follow the Wahls dietary strategy may additionally benefit from ensuring adequate iron intake through food sources such as oysters or liver. 

With combined intake from food and supplements, excessive intakes of several micronutrients are of concern. In both groups, a high proportion with excessive intake was observed for vitamin D and niacin, and for magnesium in the Swank group. As part of the intervention of this study, vitamin D doses were adjusted on the basis of serum laboratory values, and therefore it is uncertain how generalizable the excessive vitamin D intake from food and supplements is in this study. In the healthy population, prolonged excessive intake may increase the risk for micronutrient toxicity; however, it is possible that the chronic inflammation and immunologic activity associated with MS increases micronutrient needs of people with MS. Future studies are needed to evaluate micronutrient status with micronutrient biomarkers to determine the specific micronutrient requirements of pwMS.

This study is strengthened by the use of 3-day weighed food records, robust analytical methods, and high diet adherence reported previously as 87% and 80% at 12 weeks for the Swank and Wahls diets, respectively [19]. However, there are also several limitations to this study. First, the use of weighed food records collected on consecutive days may bias our data due to the influence of food choices one day on the next (e.g., leftovers eaten the following day). Second, the sample size in this study is smaller than typically recommended for the NCI method (*n* = 50) to adjust usual intake; however, dietary data were collected on more days than necessary for the NCI method to help reduce variation caused by the lack of sample size. Finally, laboratory evaluation of micronutrient status with biomarkers was not conducted for nutrients included in this analysis other than vitamin D, and therefore we were unable to determine if inadequate or excessive intake of micronutrients in this study was associated with deficiency or toxicity among pwMS following the Swank or Wahls diets. 

## 5. Conclusions

There is great interest among the people with MS and researchers alike for the use of specialized diets as adjunct therapy to improve quality of life and reduce fatigue; however, the restrictive nature of several of these specialized diets causes concern for risk of micronutrient deficiencies. This study is one of the first major attempts to evaluate the intake of micronutrients of diets specialized for MS. These results indicate that compared to the usual diet of people with RRMS, the Swank and Wahls diets are associated with reductions in the proportion with inadequate intake for several micronutrients, especially with intake from supplements, for which deficiency may be associated with symptom burden including magnesium and the vitamins A, C, D, and E. 

## Figures and Tables

**Table 1:** Baseline characteristics of participants who completed a 12-week intervention of the Swank or Wahls diets ^1^.

Characteristics	Swank	Wahls	*p*-Value ^2^
*n*	38	39	-
Age (years)	46.9 ± 1.7	46.4 ± 1.5	0.84
Gender (female)	35 (92.1)	32 (82.1)	0.31
MS duration (years)	12.1 ± 1.6	9.3 ± 1.0	0.14
Disease modifying drug use			0.83
None	13	10	
Oral	11	11	
Injectable	10	12	
Infused	4	6	
Race (Caucasian)	36 (94.7)	38 (97.4)	0.99
Education			0.32
High school	0 (0.0)	3 (7.7)	-
Some college	12 (31.6)	10 (25.6)	-
4-year degree	11 (28.9)	8 (20.5)	-
Advanced degree	15 (39.5)	18 (46.2)	-
Smoking status			0.13
Never	29 (76.3)	23 (59.0)	-
Former	3 (7.9)	2 (5.1)	-
Current	6 (15.8)	14 (35.9)	-
Alcohol drinks per month ^3^			0.99
None	6 (15.8)	7 (17.9)	-
Within recommendations	29 (76.3)	29 (74.4)	-
Above recommendations	3 (7.9)	3 (7.7)	-
BMI (kg/m^2^)	27.6 ± 0.9	30.2 ± 1.3	0.11

^1^ Data are shown as mean ± SEM or N (%). ^2^ Significance determined by Fisher’s exact test or generalized linear models. ^3^ Alcohol recommendations defined as ≤1 and ≤2 standard drinks females and males, respectively. Adapted from Wahls et al. (2021) [19].

**Table 2:** Description of the low-saturated fat (Swank) and modified Paleolithic elimination (Wahls) diets ^1^.

Diet Component	Swank	Wahls
Energy, kilocalories	Adjust to meet energy needs	Adjust to achieve and maintain a healthy body mass index
Fruits and vegetables, cup equivalents per day	2+ fruit 2+ vegetables	2–3+ dark green leafy vegetables 2–3+ sulfur-rich vegetables 2–3+ deeply colored fruits and vegetables White fruits and vegetables limited Nightshade vegetables/spices avoided
Protein, ounces per day	Adequate in quantity and quality - Beef, pork, organ meat, egg yolks, and dark meat poultry and skin avoided - White meat poultry, white fish, shellfish, and egg whites as desired - ≤1.75 fatty fish - Nuts and legumes allowed if low in saturated fat	6–12+ meat/fish - Beef, pork, poultry, game, fish, shellfish, organ meat as desired - Eggs and legumes not allowed - 16 fatty fish per week - 12 organ meat per week - 4 nuts, soaked and rinsed
Grains, ounce equivalents per day	4, whole grains preferred	Not allowed
Dairy, cup equivalents per day	2 if <1% dairy fat	Cow, goat, mare, soy not allowed
Fats	- ≤15 g saturated fat per day - 20–50 g oil per day	As desired for satiety and weight maintenance - Clarified butter, animal fats, coconut oil, avocado oil, extra virgin olive oil, sesame oil, sunflower oil as desired - ≤30 g flax, hemp, and walnut oil per day - All other fats and oils not allowed
Sweeteners	Minimal for taste	≤1tsp allowed sweeteners per day
Salt	As desired	As desired
Alcohol, standard drinks per day	1 wine or mixed drink in evening	≤1 for women, ≤2 for men
Caffeine	≤3 cups caffeinated beverages per day	As desired
Seaweed, algae, nutritional yeast, fermented foods	No recommendation, must be low in saturated fat	1 serving each per day

^1^ Adapted from Wahls et al. (2019) [7].

**Table 3:** Proportion with inadequate micronutrient intake from food and supplements at baseline and after adapting the Swank or Wahls diets among individuals with RRMS ^1,2^.

	Swank			Wahls		
Micronutrient	Baseline	12 Weeks	24 Weeks	Baseline	12 Weeks	24 Weeks
**Vitamins**						
Vitamin A	19.3	2.2 *	1.4 *	30.9	0.8 *	0.0 *
Vitamin C	28.6	2.9 *	2.6 *	29.6	0.7 *	1.4 *
Vitamin D	27.7	2.8 *	0.7 *	16.9	1.0 *	0.3 *
Vitamin E	31.9	2.5 *	0.5 *	38.7	0.3 *	0.4 *
Thiamin	9.6	2.8	0.0 *	15.9	2.8 *	0.8 *
Riboflavin	6.2	2.6	0.0	12.5	0.8 *	0.2 *
Niacin	2.7	1.6	0.0	5.9	0.0	0.0
Vitamin B_6_	17.1	4.2 *	3.9 *	24.3	0.8 *	3.9 *
Folate	9.9	2.1	0.5	19.7	0.3 *	0.2 *
Vitamin B_12_	14.0	13.4	12.6	28.6	0.1 *	0.1 *
**Minerals**						
Calcium	33.7	32.6	38.1	35.9	60.7 *	55.4 *
Copper	12.6	2.7	0.2 *	7.8	0.0	0.1
Iron	11.5	14.4	11.4	11.5	21.4	19.2
Magnesium	32.2	11.5 *	6.5 *	44.9	7.9 *	12.8 *
Phosphorous	3.4	4.6	1.7	5.5	1.3	1.3
Selenium	3.4	1.7	0.1	6.2	0.0	0.0
Zinc	14.6	1.9 *	0.4 *	19.5	0.0 *	0.2 *

^1^ Data are shown as percentages. ^2^ Within-group statistical significance determined by one proportion *z*-tests. * Indicates significant difference compared to corresponding baseline value.

**Table 4:** Proportion with inadequate micronutrient intake from food at baseline and after adapting the Swank or Wahls diets among individuals with RRMS ^1,2^.

	Swank			Wahls		
Micronutrient	Baseline	12 Weeks	24 Weeks	Baseline	12 Weeks	24 Weeks
**Vitamins**						
Vitamin A	20.7	19.8	36.6 *^,^**	44.3	13.1 *	2.3 *
Vitamin C	44.6	19.8 *	24.1 *	50.0	1.5 *	9.3 *^,^**
Vitamin D	96.3	82.3 *	77.6 *	99.9	86.6 *	87.8 *
Vitamin E	50.1	32.3 *	37.0	56.1	26.7 *	34.0 *
Thiamin	11.5	8.7	5.2	5.7	13.6 *	22.8 *
Riboflavin	5.3	4.5	1.9	4.5	5.3	7.8
Niacin	2.8	2.4	0.0	5.3	0.0	0.0
Vitamin B_6_	23.1	11.3	4.8 *	15.3	7.1	5.7
Folate	21.6	20.9	15.9	26.4	16.1	26.9
Vitamin B_12_	14.0	13.9	24.3	5.3	17.4 *	14.6 *
**Minerals**						
Calcium	47.4	51.7	59.5	54.7	86.5 *	75.5 *
Copper	8.7	9.9	3.4	6.4	0.1	1.4 **
Iron	12.6	14.4	11.7	18.3	23.0	19.7
Magnesium	37.7	26.7	22.2	50.8	28.5 *	31.1 *
Phosphorous	3.5	4.7	1.8	5.6	1.3	1.3
Selenium	2.7	2.9	1.8	6.3	2.3	0.3
Zinc	17.4	25.2	21.7	12.5	11.8	5.0

^1^ Data are shown as percentages. ^2^ Within-group statistical significance determined by one proportion *z*-tests. * Indicates significant difference compared to corresponding baseline value, and ** indicates significant difference compared to corresponding 24-week value.

**Table 5:** Proportion with excessive micronutrient intake from food and supplements at baseline and after adapting the Swank or Wahls diets among individuals with RRMS ^1,2^.

	Swank			Wahls		
Micronutrient	Baseline	12 Weeks	24 Weeks	Baseline	12 Weeks	24 Weeks
**Vitamins**						
Vitamin A	1.2	0.0	0.0	0.1	0.1	0.1
Vitamin C	0.0	0.0	0.0	4.6	5.5	2.5
Vitamin D	20.8	71.9 *	55.4 *^,^**	33.9	72.2 *	76.4 *
Vitamin E	0.0	0.0	0.0	0.0	0.0	0.0
Niacin	5.5	44.8 *	44.8 *	8.6	30.6 *	6.5 **
Vitamin B_6_	1.9	0.0	0.0	2.8	2.8	2.5
Folate	4.5	9.7	9.8	0.3	0.0	0.0
**Minerals**						
Calcium	1.1	1.0	3.0	0.0	0.0	0.4
Copper	0.0	0.0	0.0	0.0	0.5	0.0
Iron	4.9	2.9	6.2	9.3	0.1	0.1
Magnesium	2.6	2.6	10.7 *^,^**	0.9	0.2	0.3
Phosphorous	0.0	0.0	0.0	0.0	0.0	0.0
Selenium	0.0	0.1	0.1	0.0	0.0	0.0
Zinc	4.2	0.3	4.4 **	0.4	0.0	1.4

^1^ Data are shown as percentages. ^2^ Within-group statistical significance determined by one proportion *z*-tests. * Indicates significant difference compared to corresponding baseline value, and ** indicates significant difference compared to corresponding 24-week value.

## Data Availability

The data presented in this study are available on request from the corresponding author. The data are not publicly available due to the privacy of the study participants.

## Trial Registration

Clinicaltrials.gov Identifier: NCT02914964.
